# Dimensional Changes of Tracheids during Drying of Radiata Pine (*Pinus radiata* D. Don) Compression Woods: A Study Using Variable-Pressure Scanning Electron Microscopy (VP-SEM)

**DOI:** 10.3390/plants7010014

**Published:** 2018-02-27

**Authors:** Miao Zhang, Bronwen G. Smith, Brian H. McArdle, Ramesh R. Chavan, Bryony J. James, Philip J. Harris

**Affiliations:** 1 School of Biological Sciences, The University of Auckland, Private Bag 92019, Auckland Mail Centre, Auckland 1142, New Zealand; mzha084@aucklanduni.ac.nz (M.Z.); r.chavan@auckland.ac.nz (R.R.C.); 2 School of Chemical Sciences, The University of Auckland, Private Bag 92019, Auckland Mail Centre, Auckland 1142, New Zealand; b.smith@auckland.ac.nz; 3 Department of Statistics, The University of Auckland, Private Bag 92019, Auckland Mail Centre, Auckland 1142, New Zealand; b.mcardle@auckland.ac.nz; 4 Department of Chemical & Materials Engineering, The University of Auckland, Private Bag 92019, Auckland Mail Centre, Auckland 1142, New Zealand; b.james@auckland.ac.nz

**Keywords:** mild compression wood, severe compression wood, shrinkage, swelling, environmental SEM, tracheid cell walls

## Abstract

Variable-pressure scanning electron microscopy was used to investigate the dimensional changes in longitudinal, tangential and radial directions, on wetting and drying, of tracheids of opposite wood (OW) and three grades of compression woods (CWs), including severe CW (SCW) and two grades of mild compression wood (MCW) (MCW1 and MCW2) in corewood of radiata pine (*Pinus radiata*) saplings. The CW was formed on the underside and OW on the upper side of slightly tilted stems. In the longitudinal direction, the shrinkage of SCW tracheids was ~300% greater than that of OW tracheids, with the shrinkage of the MCW1 and MCW2 tracheids being intermediate. Longitudinal swelling was also investigated and hysteresis was demonstrated for the tracheids of all corewood types, with the extent of hysteresis increasing with CW severity. A statistical association was found between longitudinal shrinkage and the content of lignin and galactosyl residues in the cell-wall matrix. The galactosyl residues are present mostly as (1→4)-β-galactans, which are known to have a high capacity for binding water and swell on hydration. The small proportions of (1→3)-β-glucans in the CWs have similar properties. These polysaccharides may play a functional role in the longitudinal shrinking and swelling of CW tracheids. Tangential shrinkage of tracheids was greater than radial shrinkage but both were greatest for OW and least for SCW, with the MCW1 and MCW2 being intermediate.

## 1. Introduction

In coniferous gymnosperms (softwoods), compression wood (CW) is a reaction wood formed when growing stems are tilted from the vertical as the result of various factors such as the weight of snow or the slope of the land. CW forms on the lower side of tilted stems to correct the growth to the vertical [1]. The wood formed on the opposite side of the stem is referred to as opposite wood (OW) and is very similar in structure and composition to normal wood (NW). Although CW formation is beneficial to the tree, CW is regarded as a defect in the timber industry. When wood is dried and rewetted, it shrinks and swells and this dimensional instability is a major obstacle in the use of wood, especially for structural purposes [2]. CW shows particularly high longitudinal shrinkage and swelling [1]. Because of the differences in longitudinal shrinkage and swelling between CW and OW or NW, where these wood types occur adjacent to one another in timber, severe warping can occur with a loss of value [2]. CW also shows differences from OW and NW in tangential and radial shrinkage but the differences are much less pronounced [1].

During drying, wood shrinkage occurs only when all the free water, which is water in the lumens of the cells or in intercellular spaces, has been removed, a point known as the fibre saturation point. Wood shrinkage and swelling depend on the amount of bound water, which is water adsorbed within the cell walls. Thus, cell walls play a pivotal role in these processes [3,4]. In softwoods, the cell walls are mostly of tracheids because softwoods are composed predominantly of this cell type [5]. Tracheids are thick-walled, elongated cells that in CW are shorter and have thicker walls than in OW and NW. As in all plant cell walls, these walls consist of a fibrillar phase in the form of cellulose microfibrils set in a matrix phase. In NW and OW, the tracheid walls consist of a thin primary wall and a thick secondary wall composed of three layers (S1, S2 and S3), with the middle layer (S2) being the thickest. The angle the cellulose microfibrils make to the vertical axis of the tracheid in the S2 layer is known as the cellulose microfibril angle (MFA). In stems of the commercially important coniferous gymnosperm *Pinus radiata* (radiata pine), two regions of wood have been recognized: corewood, which is formed in the first ten growth rings from the pith; and outerwood, which is formed further out [6]. In the outerwood, the MFA in tracheid walls of CW is higher than in tracheid walls of NW or OW and is considered to be related to the high longitudinal shrinkage of CW [7]. In corewood, the MFA in tracheid walls of NW or OW is already high (>~35°) and is the same or similar to that in tracheid walls of CW [8,9]. Thus, even though a high MFA may be necessary for high longitudinal shrinkage of CW, it is not sufficient and polymers in the cell-wall matrix are likely to play an important role [10].

The cell-wall matrix of NW and OW tracheids of gymnosperms, including *P. radiata*, are composed of the non-cellulosic polysaccharides heteromannans (*O*-acetyl-galactoglucomannans) and smaller proportions of heteroxylans [arabino(4-*O*-methylglucurono)xylans], in addition to the aromatic polymer lignin composed mostly of guaiacyl (G) units, with only trace amounts of *p*-hydroxyphenyl (H) units [8,11]. In comparison, the cell-wall matrix of CW tracheids contains smaller proportions of heteromannans and heteroxylans but larger proportions of lignin containing significant proportions of H-units. Additionally, the cell-wall matrix of CW tracheids contains (1→4)-β-galactans (~10%) and small proportions of (1→3)-β-glucans [8,12,13]. Positive statistical associations have been found between longitudinal shrinkage and the content of (1→4)-β-galactans in CW in the corewood of *P. radiata* saplings [8]. A similar association has also been found between longitudinal shrinkage and lignin content. Thus, positive associations have been found between longitudinal shrinkage of CW and two cell-wall matrix components.

As described above, CW is more accurately referred to as severe CW (SCW) but it is increasingly being recognized that a continuum of CWs occur between SCW and NW or OW, with the intermediate grades, being referred to as mild CWs (MCWs) [14,15]. These MCWs have been much less studied than SCW. However, we recently reported two studies on two grades of MCWs, MCW1 and MCW2, as well as SCW and OW, in stem corewood of *P. radiata* saplings [12,13]. The saplings were tilted from the vertical to induce the formation of CW but only a small tilt angle (~8–20°) was used to try to maximize MCW formation relative to SCW. The different wood types were identified based on the distribution of lignin in the tracheid cell walls in transverse sections. This was determined by the distribution of lignin autofluorescence using UV fluorescence microscopy [16]. In OW tracheids, the lignin was mostly in the S3 layer of the secondary wall, in the compound middle lamella (middle lamella plus primary cell walls) and in the middle lamella in the cell corners. In MCW1 tracheids, some lignin was also present at the cell corners in the outer region of the S2 layer (S2L region). In MCW2 tracheids, lignin in this outer region of the S2 layer (S2L region), was present all around the cells. In SCW tracheids, greater amounts of lignin were in this S2L region and there was no lignin in the CML at any position. The cells also became more circular in transverse section in going from OW to SCW. A S3 secondary wall layer was present in OW tracheids and a very thin one was also present in MCW1 tracheids but not in MCW2 or SCW tracheids. SCW was the only wood type with tracheid walls having helical cavities in the inner region (S2i region) of the S2 layer.

All our studies were carried out on small discs of wood, 0.5 mm in diameter that were all of a specific wood type, either OW, MCW1, MCW2 or SCW [12,13]. This permitted very accurately defined wood types to be used although only small amounts of wood were available for the investigations. We had earlier reported that there were no significant differences between the high cellulose MFAs in tracheid cell walls of SCW, OW and NW in stem corewood of *P. radiata* saplings of similar age [8]. We therefore focused on tracheid cell-wall matrix polymers, rather than cellulose, because of their likely importance in longitudinal shrinkage of compression corewoods. The lignin content was found to increase with CW severity, with OW, MCW1, MCW2 and SCW containing 26%, 28%, 32% and 35% lignin respectively. The H-unit content of the lignin also increased with CW severity, with OW, MCW1, MCW2 and SCW containing >1%, 7%, 11% and 14%, respectively and the H/G ratio increased in the same order [13]. Immunofluorescence and immunogold microscopy with monoclonal antibodies was used to specifically determine the distributions of (1→4)-β-galactans and (1→3)-β-glucans in the tracheid cell walls. This showed that the (1→4)-β-galactans occurred as a band in the outer region of the S2 wall layer (S2L region) of the tracheid walls in all three grades of CW, with the band becoming wider and more intense in the order MCW1, MCW2 and SCW. The (1→4)-β-galactans thus co-located with lignin in CW tracheids. There was only extremely weak labelling of the tracheid walls in OW, probably of the primary walls. As with the (1→4)-β-galactans, the (1→3)-β-glucans also occurred as a band in the S2 wall layer of CW tracheids, which became wider and more intense with CW severity. However, the (1→3)-β-glucans were located in the inner region (S2i region) of the S2 wall layer. Differences between the wood types were also found in the neutral-monosaccharide compositions of the non-cellulosic polysaccharides. In particular, the proportion of galactose was consistent with the immunolabelling results for (1→4)-β-galactans, with the lowest in OW, the highest in SCW and intermediate proportions in MCW1 and MCW2.

In the present study, we used similar, small discs cut from the same four corewood types and determined the longitudinal shrinkage and swelling of the tracheids, as well as their radial and tangential shrinkage, by variable-pressure scanning electron microscopy (VP-SEM), sometimes referred to as environmental scanning electron microscopy (ESEM). VP-SEM is an excellent technique for this purpose because the samples can be imaged without pre-treatment. The samples can be hydrated (adsorption) and dehydrated (desorption) by altering the water vapour pressure and hence the relative humidity in the chamber [17]. The technique has been used to study tracheid shrinkage in earlywood and latewood of Norway spruce (*Picea abies*) but CW has not been examined [18]. We hypothesized that, based on the composition of the cell-wall matrix polymers, the longitudinal shrinkage and swelling of the tracheids in the MCW1 and MCW2 would be intermediate between those in OW and SCW.

## 2. Results

### 2.1. Longitudinal Shrinkage and Swelling of the Tracheids Increases with CW Severity and Shows Hysteresis

**Figure 1:**
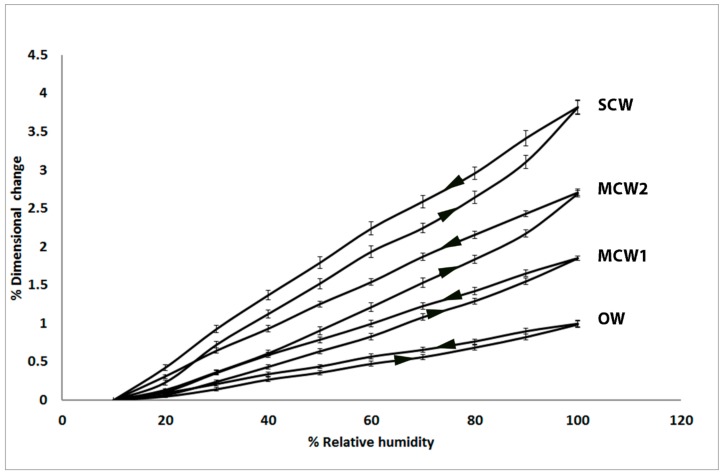
Plot showing the percentage longitudinal dimensional changes at the 10 desorption (from 100% to 10% RH) and 9 absorption steps (from 10% to 100% RH) in 10% steps of tracheids of the four corewood types (SCW, MCW2, MCW1 and OW) in Tree 1. The SCW showed the greatest change, OW the least and MCW1 and MCW2 were intermediate. The shrinkage and swelling of all the corewood types was reversible. Considerable hysteresis was shown between swelling and shrinkage for all the corewood types and the dimensional changes were always greater during desorption than absorption for any given RH.

Longitudinal dimensional changes at the 10 desorption and 9 absorption steps of the tracheids (measured as dimensional changes in the longitudinal walls) of the four corewood types in Tree 1 are shown in Figure 1. Non-linear relationships were observed between relative humidity and percentage dimensional change and for all the corewood types, with SCW showing the greatest change, OW the least and MCW1 and MCW2 intermediate. There was considerable hysteresis observed between swelling and shrinkage for all the corewood types and the closing of all the hysteresis loops indicated that shrinkage and swelling were reversible. For any given relative humidity (RH), the dimensional changes were always greater during desorption than adsorption and SCW and MCW 2 showed more pronounced hysteresis than MCW 1 and OW.

Longitudinal shrinkage but not swelling, of tracheids of the four corewood types in Trees 2 and 3 was also determined and the percentages were similar to those obtained in Tree 1 (Table 1). For each of the trees, the percentage shrinkage increased from OW, which showed the least percentage shrinkage (~0.95%), to SCW, which showed the most (~3.8%), with the increase following the CW severity (Table 1). This represented an increase of ~300% increase in shrinkage from OW to SCW. Statistical analysis of these results and those of tangential and radial shrinkage of the tracheids is shown below.

### 2.2. Tangential and Radial Shrinkage of the Tracheids Decreases with CW Severity, with Tangential Shrinkage Greater than Radial Shrinkage

The tangential and radial shrinkage of tracheids in the four corewood types of Trees 1, 2 and 3 is also shown in Table 1. Unlike the longitudinal shrinkage, both the tangential and radial shrinkage of the tracheids in each corewood type decreased with increasing CW severity. The OW tracheids had the highest percentage shrinkage in both the tangential and radial directions and the SCW tracheids had the least. The percentage tangential and radial shrinkage of MCW was intermediate between that of SCW and OW, with MCW1 showing more shrinkage than MCW2. Tangential shrinkage of the tracheids was approximately twice that of the radial shrinkage in all the corewood types. The tangential and radial shrinkage of OW was ~6.6% and ~3.1% and of SCW was ~4.2% and ~2.1%, representing decreases in shrinkage of ~37% and 32%, respectively. The shrinkage of the tracheids forming the four different corewood types was thus anisotropic.

### 2.3. Statistical Analysis of Tracheid Shrinkage by Two-Way Factorial MANOVA Confirmed the Differences between Corewood Types

**Figure 2:**
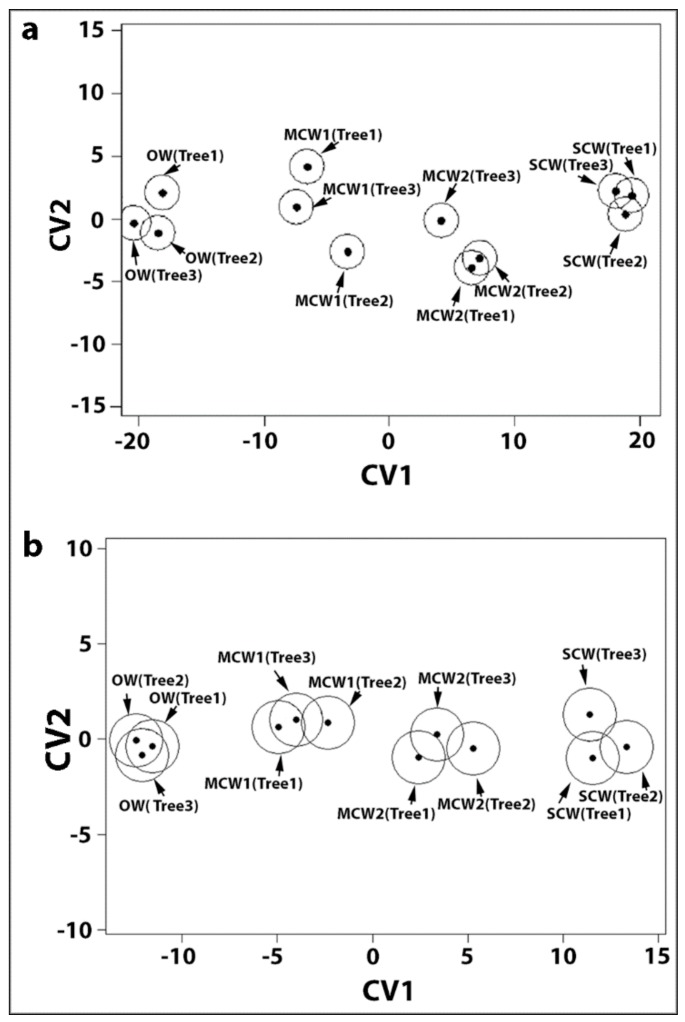
Canonical discriminant analysis (CDA) plot. (**a**) The four corewood types from three trees defined by the first two canonical variates (CV1 and CV2) obtained from CDA conducted on the shrinkage of the tracheids in the longitudinal, tangential and radial directions. The centroids for each corewood type of each tree with approximate 95% confidence regions are separated based on the differences in the shrinkage of the tracheids between corewood types and not trees. (**b**) The four corewood type from three trees defined by CV1 and CV2 obtained from CDA conducted on the tracheid cell-wall shrinkage (longitudinal shrinkage and transverse shrinkage of the radial and tangential walls). The centroids for each corewood type of each tree with approximate 95% confidence regions are separated based on the differences in the deformation of the tracheids on drying between corewood types and not trees.

The effects of corewood type and tree number on the tracheid longitudinal, tangential and radial shrinkage was examined by a two-way factorial MANOVA. This showed a highly significant effect of corewood type (*p* = 6.89 × 10^−11^) and a somewhat less significant effect of tree number (*p* = 2.77 × 10^−10^). However, the situation was complicated by clear evidence that the differences between the trees depends on the corewood type (interaction *p* = 1.04 × 10^−4^). The canonical discriminant analysis (CDA) plot (Figure 2a) shows that the four corewood types line up on a single axis (canonical variate 1, CV1), with the SCW at the positive end being associated with high values of longitudinal and low values of tangential and radial shrinkage and with the OW at the negative end being associated with low values of longitudinal and high values of tangential and radial shrinkage. The confidence ellipses on each point emphasises how separate they are. The correlation coefficients of all the variables for CV1 and CV2 are listed in Table 2.

### 2.4. Micrographs of Transverse Surfaces Show that Tracheids Become Less Well Ordered, More Rounded and Their Walls Thicker with CW Severity

**Figure 3:**
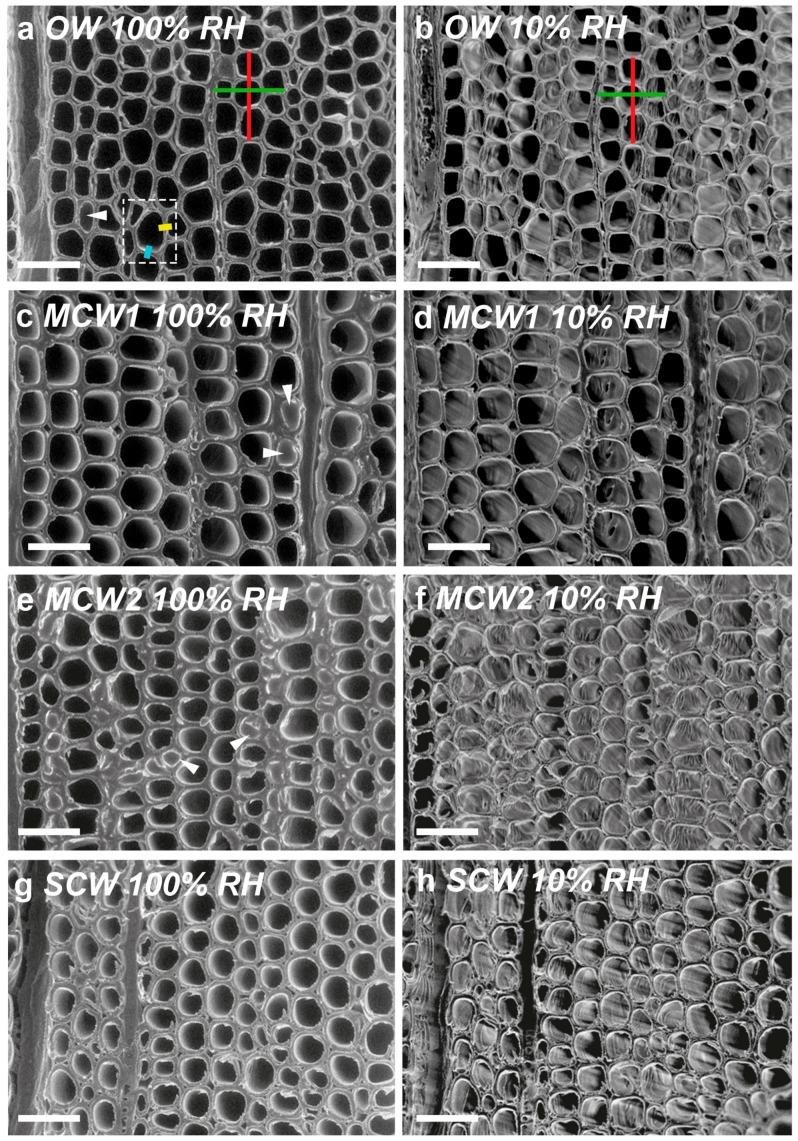
VP-SEM micrographs of cut transverse surfaces of discs showing the tracheids from the four corewood types in Tree 1. (**a**) OW at 100% RH, (**b**) OW at 10% RH, (**c**) MCW1 at 100% RH, (**d**) MCW1 at 10% RH, (**e**) MCW2 at 100% RH, (**f**) MCW2 at 10% RH, (**g**) SCW at 100% RH, (**h**) SCW at 10% RH. At 100% RH, free water fills some of the tracheid lumens (arrow heads). OW tracheids have the thinnest walls but the largest lumen diameters. The wall thickness increases and the lumen diameter decreases with increasing CW severity. The tracheids in all four corewood types are better aligned radially than tangentially. Shrinkage in the radial direction (red line in (**a**)) and in the tangential direction (green line in (**a**)) was investigated by measuring the dimensional changes between two reference points spanning three or four tracheids. Transverse wall shrinkage was also measured of tangential walls (light blue line) and of radial walls (yellow line) was also measured; the thickness of the double wall of two adjacent tracheids was measured. Scale bar = 50 µm.

As shown in the micrographs of the transverse surfaces of discs of the four corewood types (Figure 3), the tracheids are all well-ordered in the radial direction but are very much less ordered in the tangential direction. As the CW severity increases, the shapes of the tracheids in transverse view gradually changes from the rectangular or polygonal shape of the OW tracheids (Figure 3a,b) to the more rounded or oval shape of the SCW tracheids (Figure 3g,h). The thicknesses of the tracheid walls and the lumen diameters also change with severity of the CW. These were quantified on micrographs and the thicknesses of the radial and tangential tracheid walls in a particular corewood type were not significantly different (*p* > 0.05) but both increased with CW severity (Table S1). Lumen diameters were significantly greater in the radial direction than the tangential (*p* < 0.01) but both diameters decreased with CW severity. Tracheids in the OW had the thinnest walls and largest lumens, whereas those in the SCW had the thickest walls and the smallest lumens.

### 2.5. Transverse Wall Shrinkage Decreased with CW Severity and was Greater for Radial Walls than Tangential Tracheid Walls

Comparison of micrographs of exactly the same transverse surface areas at 100% and 10% RHs show differences, mostly associated with the presence of free water in the 100% RH micrographs. For example, some of the tracheids contained free water (see arrows in Figure 3). However, at 10% RH, all the free water and most of the bound water was removed. Changes as a result of drying were quantified on micrographs (Table S1). The lumen diameters of the tracheids showed the greatest shrinkage in OW and least in SCW, with shrinkage of the tangential diameters being greater for a given corewood type than of the radial diameters. Transverse tracheid wall shrinkage, determined from changes in wall thickness (width), was greatest in the OW and least in the SCW, with shrinkage of the radial walls being greater for a given corewood type than the tangential walls. Shrinkage of the radial and tangential walls of OW was ~4.7% and ~2.14% and of SCW ~2.0% and ~1.1%, representing differences between the two corewood types of ~58 and 48%, respectively. For all the corewood types, tangential shrinkage was about twice that of radial.

The effects of corewood type and tree number on longitudinal and transverse tracheid wall shrinkage was also examined statistically by two-way factorial MANOVA. The results were similar to the MANOVA shown above for overall tracheid shrinkage, with the corewood type showing a highly significant effect (*p* = 9.61 × 10^−7^) but the between tree variation is much less (*p* = 0.0067). The CDA plot is shown in Figure 2b and the correlation coefficients of all the variables for CV1 and CV2 are listed in Table 2.

### 2.6. Canonical Correlation Analysis Showed that Longitudinal Tracheid Shrinkage is Positively Correlated with Lignin Content and Galactose Percentage

**Figure 4:**
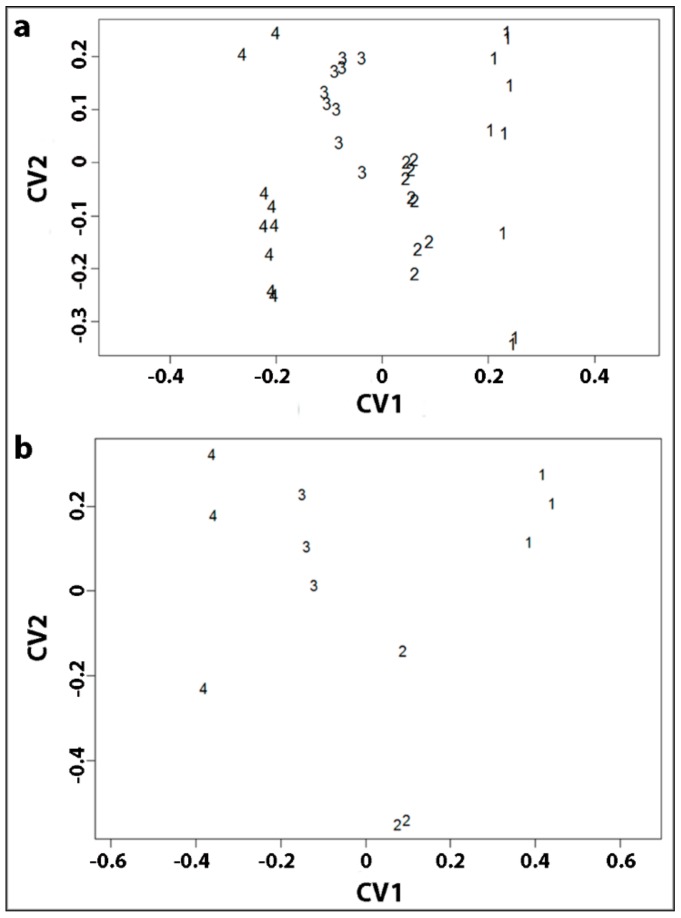
The canonical variate plot (**a**) Plot showing the separation of the four corewood types (1, OW; 2, MCW1; 3, MCW2; 4, SCW) from three trees defined by the first two canonical variates (CV1 and CV2) obtained from canonical correlation analysis conducted on a combination of tracheid shrinkage in the three directions (longitudinal, tangential and radial) and the chemical data (lignin content; percentage arabinose, galactose, xylose and mannose). (**b**) Plot showing the separation of the four corewood types from three trees defined by the first two canonical variates (CV1 and CW2) obtained from canonical correlation analysis conducted on a combination of tracheid wall shrinkage (longitudinal shrinkage and transverse shrinkage of the radial and of the tangential walls) and the chemical data (lignin content; percentage arabinose, galactose, xylose and mannose).

To investigate the relationship between tracheid shrinkage (longitudinal, tangential and radial) of the four corewood types and their cell-wall compositions, a canonical correlation analysis was conducted. In the overall canonical correlation analysis, the association between the shrinkage variables set and the chemical variables set was assessed by establishing the canonical correlation coefficient for each pair of linear composites derived from the data. The analysis yielded three functions with canonical correlations of 0.992, 0.468 and 0.164, with only the first two functions being considered noteworthy in the context of this study (99.2% and 46.8% of the shared variance, respectively). This suggested a strong linear relationship between the shrinkage variables set and the chemical variables set. To determine the most important variables in a given pair of canonical variates, the canonical variate scores were analysed (Table 3). The structure coefficients [19] of the first variate (CV1) indicated that the longitudinal shrinkage is positively associated with lignin content and percentage galactose (in an acid hydrolysate) but negatively associated with arabinose, xylose and mannose percentages. Tangential and radial shrinkage were both negatively associated with the lignin contents and galactose percentages but positively associated with arabinose, xylose and mannose percentages. Neither the CV 2 nor the CV 3 correlations are particularly large for both sets of variables and so these canonical variates yielded little information about the data and will not be considered further. To see the relationship, a canonical variate plot was generated (1, OW; 2, MCW1; 3, MCW2; 4, SCW) (Figure 4a) based on an analysis of the combined variables in the two sets of data. This plot indicated considerable separation among the four corewood types along the first canonical variate.

A second, related canonical correlation analysis was conducted to specifically investigate the relationship between tracheid cell-wall shrinkage (longitudinal and transverse of the radial and of tangential walls) and chemical composition. The longitudinal tracheid wall shrinkage was, of course, the same as the longitudinal tracheid shrinkage, which has already been considered. As in the first analysis, this analysis yielded three functions with canonical correlations of 0.998, 0.787 and 0.522, which suggested a strong linear relationship between the two sets of variables. The canonical variate scores were further analysed (Table 3), with similar results being obtained to those in the first analysis, with longitudinal tracheid wall shrinkage, unsurprisingly, being positively associated with the lignin contents and the galactose percentages but negatively associated with arabinose, xylose and mannose percentages. The transverse wall shrinkage of both the radial and tangential tracheid walls was negatively associated with lignin contents and galactose percentages but positively associated with arabinose, xylose and mannose percentages. As above, a canonical variate plot was generated to show the considerable separation among the four corewood types along the first canonical variate (Figure 4b).

### 2.7. Diameters of Tracheid Bordered Pits Did Not Change Significantly on Drying but the Diameters of the Pit Apertures Decreased with Increasing CW Severity

**Figure 5:**
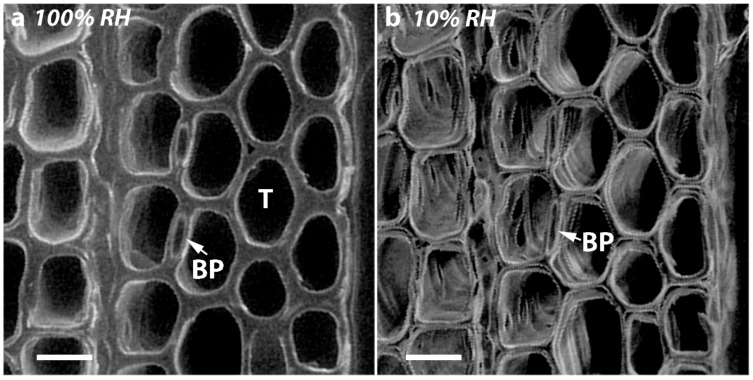
VP-SEM micrographs showing the cut transverse surface of a disc of MCW1 from Tree 1 (**a**) at 100% RH and (**b**) at 10% RH. The chamber (arrow) of the tracheid (T) bordered pits (BP) has collapsed on drying. Scale bar = 20 µm.

The diameters of the bordered pits and their apertures in the tracheids of the different corewood types of Tree 1 are shown in Table 4, which also shows their percentage changes on drying. One-way ANOVA and the post-hoc Duncan’s test were used to statistically compare the diameters of the bordered pits and their apertures in the different corewood types and before and after drying (i.e., ongoing from 100% RH to 10% RH) (Table 5). Before drying, the bordered pits of OW and MCW1 had similar diameters (*p* > 0.1), which were larger than those of the bordered pits of MCW2 (*p* < 0.05) and SCW had the smallest diameter bordered pits (*p* < 0.001) (Table 4). The apertures of the bordered pits of SCW were also the smallest of the different corewood types (*p* < 0.001). On drying, the diameters of the bordered pits of the different corewood types showed little change. However, the diameters of the pit apertures decreased with increasing CW severity. The percentage decrease was greatest in the SCW (4.69%) and least in the OW (1.61%), with MCW1 and MCW2 intermediate. There were significant differences in the changes of the pit aperture diameters on drying between the four corewood types (*p* < 0.001), in which the differences between the MCW2 and MCW1 were less detectable (*p* < 0.05) (Table 5). Comparison of micrographs of transverse surfaces of MCW1 at 100% and 10% RH showed the chambers of the bordered pits collapsed during drying (Figure 5). This collapse probably accounts, at least in part, for the decrease in aperture diameter after drying.

**Figure 6:**
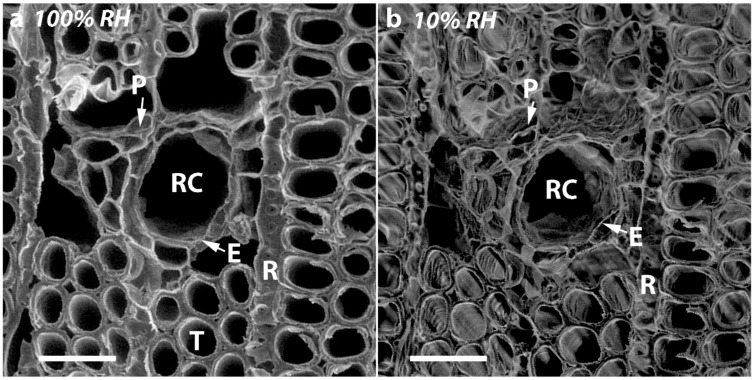
VP-SEM micrographs of the cut transverse surface of a disc of SCW from Tree 1 (**a**) at 100% RH and (**b**) at 10% RH. The structure of the resin canal (RC) and associated cell types change on drying. The thin-walled epithelial cells (E) around the resin canal and parenchyma cells (P) are collapsed after drying to 10% RH. R = ray tracheid. T = Tracheid. Scale bar = 50 µm.

**Figure 7:**
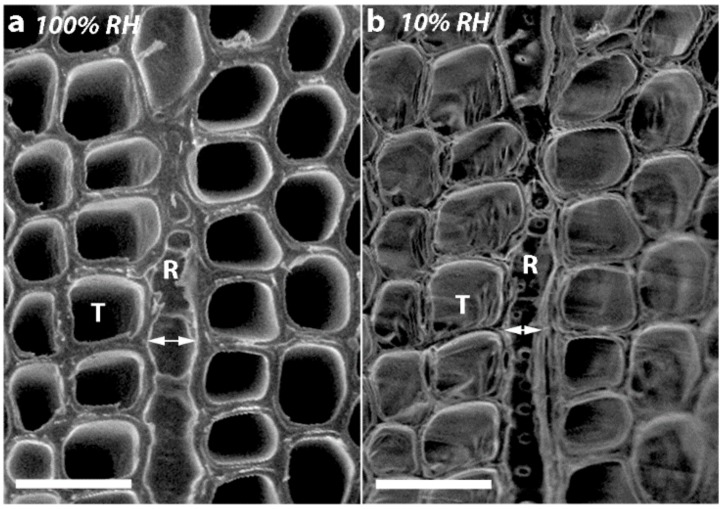
VP-SEM micrographs showing the cut transverse surface of a disc of MCW1 from Tree 1 (**a**) at 100% RH and (**b**) at 10% RH. The thin-walled, radial ray tracheid (R) shrank tangentially after drying to 10% RH. The distance between the two longitudinal walls of the ray tracheid (arrows) became much smaller after drying. T tracheid. Scale bar = 50 µm.

Comparison of micrographs of transverse surfaces of SCW at 100% and 10% RH also showed major changes of the resin canals and associated cell types on drying (Figure 6). The thin-walled epithelial and parenchyma cells collapsed. In addition, the thin-walled, radial ray tracheids shrank tangentially after drying and the distance between the two tangential walls of the ray tracheids (arrows) became much smaller (Figure 7).

## 3. Discussion

As we hypothesized, the present study showed that the longitudinal shrinkage of the tracheids of the two grades of MCW, MCW1 and MCW2, was intermediate between that of OW and SCW, with the shrinkage of MCW1 being less than MCW2. The percentage longitudinal shrinkage of OW and SCW tracheids was similar to that reported by others for radiata pine in which the shrinkage of small strips (e.g., 3 mm radial × 10 mm tangential × 100 mm longitudinal) of wood was measured using micrometre callipers [20,21,22]. The shrinkage was also similar to the longitudinal swelling of OW, NW and SCW reported by Brennan et al. [8] also using similar strips of radiata pine corewood. Interestingly, one of these studies [22] included wood strips that contained MCW, although the size of the strips would preclude examining all the tracheids to check if they were indeed MCW. Nevertheless, the MCW strips also showed longitudinal shrinkage intermediate between SCW and NW.

Our longitudinal shrinkage results are consistent with the tracheid walls of the MCWs MCW1 and MCW2 containing intermediate proportions of (1→4)-β-galactans determined both by immunomicroscopy, which showed they were present in the S2L region of the tracheid walls and by determining the neutral monosaccharide compositions of the cell-wall matrix polysaccharides [12]. Statistically, there was a positive association between longitudinal shrinkage and the proportions of galactosyl residues as a percentage of the total neutral monosaccharides released by acid hydrolysis conditions that did not hydrolyse cellulose. Similar associations have previously been reported between longitudinal swelling and the proportions of galactose in acid hydrolysates of OW, NW and SCW of corewood of radiata pine [8] and between longitudinal shrinkage and the proportions of galactose in acid hydrolysates of logs of 24-year-old, plantation-grown loblolly pine (*Pinus taeda*) [23]. (1→4)-β-Galactans are known to have a high capacity for binding water and swell on adsorbing water (hydration) [24,25,26]. These polysaccharides may thus play a functional role in the longitudinal shrinkage and swelling of CWs.

A statistically positive association was also found in the present study between longitudinal shrinkage and lignin contents. A similar association has also been reported between lignin content and longitudinal swelling of corewood SCW [8]. However, lignin is a hydrophobic molecule and does not have a high capacity for binding water [27]. Lignin therefore probably does not play a functional role in the longitudinal shrinkage and swelling of CWs. Nevertheless, the formation and location of lignin and (1→4)-β-galactans are closely related and it is possible that this close relationship results in the statistical association. Our earlier studies showed that with increasing CW severity, the band of (1→4)-β-galactans in the S2L region of the tracheid walls increased in width and, in the same region, lignin autofluorescence also increased [12,13]. The (1→4)-β-galactans are therefore co-located with lignin and together increase in concentration with CW severity. The two components are probably covalently linked as partial delignification of radiata pine SCW resulted in increased amounts of (1→4)-β-galactans that could be extracted using 6 M sodium hydroxide [28]. This finding is consistent with the isolation of a complex containing (1→4)-β-galactans and lignin from Japanese red pine (*Pinus densiflora*) [29]. The synthesis of the (1→4)-β-galactans probably slightly precedes lignification in the S2L region, with the covalent cross links between the two components probably being formed by reactions involving quinone methide intermediates during lignin synthesis [27].

In addition to (1→4)-β-galactans, small proportions of (1→3)-β-glucans were found in all grades of CW and increase with increasing CW severity [12]. They were detected by immunomicroscopy and also by staining with pure, synthetic aniline blue fluorochrome, which specifically binds to (1→3)-β-glucans and were found to be located in the inner region of the S2 layer (S2i region) [12]. Like (1→4)-β-galactans, these polysaccharides have a high capacity for binding water and are known to swell on adsorbing water (hydration) [30,31,32]. Consequently, they may also contribute to the longitudinal shrinkage and swelling of CW. Our immunogold microscopy study [12] showed that, in SCW, these polysaccharides were located in the helical cavities in the inner region of the S2 layer. A previous immunogold microscopy study [33] also reported the same location of (1→3)-β-glucans in the tracheid walls in SCW of radiata pine and of Sitka spruce (*Picea sitchensis*) and Norway spruce (*Picea abies*). Interestingly, in the two MCWs of radiata pine, the (1→3)-β-glucans were also located in the S2i region of the tracheid walls, despite there being no cavities [12]. The presence of the glucans in the helical cavities of the SCW walls led to the suggestion they may act as sealants for these cavities [33] but the absence of cavities in the MCW walls suggests this is not their only function.

The hysteresis we found for the sorption curves for the four different corewood types has previously been reported for softwoods [4]. The bound water in tracheid walls that is responsible for the dimensional changes, is thought to be mostly hydrogen bonded to the hydroxyl groups of the non-cellulosic polysaccharides. The hysteresis being explained by it being more difficult for water to penetrate tracheid walls, with low water contents and hydrogen bond to hydroxyl groups of the non-cellulosic polysaccharides (adsorption) than for these hydrogen bonds to be broken and the water leave the walls (desorption). However, the complete recovery of the starting dimensions when the samples were “rehydrated” in the VP-SEM is consistent with the finding that all wood cell-wall hydroxyl groups were accessible to water vapour after air drying [34]. The degree of hysteresis was greater for CW and increased with CW severity. This may be related to the differences in the cell-wall matrix compositions of the different corewood types. Alternatively, it may be related to the differences in the tracheid wall thickness, which also increased with increasing CW severity. The degree of hysteresis in transverse shrinkage (tangential and radial) has previously been related to tracheid wall thickness in Norway spruce (*Picea abies*) [35].

As all the tracheids in softwoods are joined through their MLs, it is not surprising that if there is shrinkage in the longitudinal direction, there will also be dimensional changes in the tangential and radial directions. Indeed, in all the corewood types, the tracheids also showed tangential and radial shrinkage, with the tangential shrinkage being about twice that of the radial shrinkage. Similar higher values for tangential than for radial shrinkage have been reported for NW of softwood species, including radiata pine [2]. Interestingly, when the transverse surfaces of the discs were examined, the shrinkage in thickness of the radial walls, which is shrinkage in the tangential direction, was about twice that of the shrinkage in thickness of the tangential walls, which is shrinkage in the radial direction. Thus, the relative shrinkage of the tangential and radial walls appears to play a major part in determining the anisotropy of transverse shrinkage. What actually determines these shrinkage differences between the radial and tangential walls is less clear. One factor may be the presence of bordered pits in the radial but not in the tangential walls. The cellulose microfibrils in the radial walls deviate around pits, probably affecting the wall shrinkage [36]. This is consistent with the finding in our study that the diameters of the bordered pits remain almost unchanged on drying in all the corewood types. A factor that may also cause less radial than tangential shrinkage of tracheids is the arrangement of the tracheids. As was also shown in the present study, they are regularly ordered in the radial direction but less so in the tangential direction. In hardwoods, the rays have also been found to restrain shrinkage in the radial direction [2,37]. However, in the softwood *Pinus sylvestris*, cross sections of wood with and without rays showed no difference in shrinkage, suggesting they are not a limiting factor in softwood shrinkage [38].

In the present study, we found that in addition to the tracheids of SCW having a much higher (~300% higher) longitudinal shrinkage than the tracheids in OW, the tangential and radial shrinkage of the tracheids was less by ~37% and ~32%, respectively. Similar, lower tangential and radial shrinkage values for SCW than NW (or OW) have been reported for a number of softwood species [1]. Our results extend this information by showing that the tangential and radial shrinkage of the tracheids of the two mild CWs was intermediate between that of OW and SCW. In a similar way to the tangential and radial shrinkage of OW, the relative shrinkage of the tangential and radial walls appears to play a major part in determining the anisotropy of transverse shrinkage of the CWs. The lower shrinkage of the tangential and radial walls of the CWs, relative to that of OW, may result from the much greater longitudinal shrinkage driven by the presence of the (1→4)-β-galactans and possibly (1→3)-β-glucans.

## 4. Materials and Methods

### 4.1. Tree Growth and Wood Samples

In spring, seedlings of radiata pine (*Pinus radiata* D. Don) (Forest Genetics Ltd., Rotorua, New Zealand) were planted and grown outside in bags with regular irrigation at Harewood, Christchurch, New Zealand, as described by Zhang et al. [12,13]. Three saplings were used: clone 30 ramets 1 and 2 and clone 17, which are referred to as Trees 1, 2 and 3, respectively. After growing upright for six months, they were tilted by staking at ~8–20° from the vertical to produce CW and OW and harvested after another 16 months. The exact angle of tilting, measured at harvest, was for Tree 1 ~20°, Tree 2 ~13° and Tree 3 ~8°. A segment (~10 cm long) was sawn from each stem ~20 cm above the surface of the soil. The pieces were dried at 35 °C to constant weight and stored at ambient temperature. Segments (1 cm thick) were cut from each dried piece using a band saw and immersed in water at 4 °C to soften. All the stems contained one annual growth ring boundary and all the tracheids examined were of earlywood from the second year corewood.

Transverse sections (~200 µm thick) were cut from the softened segments using a sliding microtome (Model HN 40, Jung, Heidelberg, Germany). Regions within these sections containing SCW, MCW1, MCW2 and OW were identified based on the distribution of lignin in the tracheid walls as determined by its autofluorescence using fluorescence microscopy. This was done with a fluorescence microscope (model DMR; Leica, Wetzlar, Germany) equipped with an I3 filter block (excitation filter BP450–490, chromatic beam splitter 510 and emission filter LP515) [12].

Discs, each containing only one of the four corewood types, were excised from the 200-µm thick transverse sections of each tree, using a 0.5 mm Harris Uni-core^TM^ micro-puncher (ProSciTech, Kirwan QLD 4817, Australia) and the purity of each corewood type was again checked by fluorescence microscopy. Triplicate discs of each corewood type from each tree were used “as cut” or were trimmed under a stereo microscope with a double-sided razor blade as shown in Figure S1, so that they could be more easily placed on the Peltier stage to observe the tracheid radial longitudinal walls because only these walls have bordered pits (Figure S2). All the discs were kept fully saturated by immersing them in water before viewing.

### 4.2. Variable-Pressure Scanning Electron Microscopy (VP-SEM)

Discs of the four corewood types were examined using a FEI Quanta 200 FEG ESEM (FEI Company, Eindhoven, The Netherlands) used in the ESEM imaging mode with a gaseous secondary electron detector (GSED). Imaging parameters were kept constant: 10.0 KV, 4.0 spot size, 400 × magnification and the working distance was the same in all experiments. Moisture sorption experiments were done in the microscope chamber, maintained at 2 °C, with the percentage relative humidity (RH) in the chamber being controlled by altering the water vapour pressure (Table 6) [39]. 

To measure the longitudinal shrinkage and swelling of tracheids, fully water saturated, triplicate, trimmed discs of each corewood type (from Tree 1) were placed on their longitudinal surfaces on the cooled Peltier stage. The RH was adjusted from fully saturated (100% RH) to 10% RH in 10 desorption steps and then back to 100% RH in 9 adsorption steps (Table 6). The 10% RH corresponds to a ~2.6% moisture content for the wood [40]. At each step, images were taken every minute of the longitudinal surface of each of the three discs, until the distance d (~150 µm) between two references points (edges of bordered pit apertures on the same radial longitudinal wall of a tracheid) (Figure S3) on one of the discs, measured using the microscope software, remained constant (~4–5 min). Images of all three discs were then captured and the distances between similar reference points measured using eighteen tracheids (six tracheids in each of the triplicate discs) with ImageJ 1.47v software (National Institutes of Health, Bethesda, MD, USA) and the percentage dimensional changes calculated. Images from this experiment were also used to determine the shrinkage of bordered pits and their apertures by measuring the changes in the bordered pit diameters (bpd, Figure S4) and of the pit apertures (pad, Figure S4) from 100% RH to 10% RH. The diameters of thirty bordered pits and pit apertures were measured for each corewood type.

Longitudinal shrinkage but not swelling, was measured using trimmed discs of all corewood types from Trees 2 and 3 by changing the RH from 100% to 10% in only one step. Images were taken as described above. Radial and tangential shrinkage but not swelling, was also measured in a similar way except that “as cut” discs were used. The discs were placed with one of their transverse surfaces on the cooled Peltier stage. Radial and tangential shrinkage was determined by measuring the dimensional changes between two reference points spanning three or four tracheids, with the reference points being the compound middle lamella (CML) (Figure 3). The transverse surfaces of the discs were also used to measure changes on going from RH 100% to 10% in the lumen diameters of tracheids in the radial and tangential directions. These surfaces were also used to measure transverse wall shrinkage of the radial and tangential tracheid walls, by measuring the reduction in thickness (widths) of these walls (measured as double walls of adjacent tracheids). Transverse wall shrinkage of the radial and tangential walls represents shrinkage in the tangential and radial directions, respectively (Figure 3). All measurements were done on six tracheids, chosen at random, for each of the three discs. Imaging and measurements were done as described above.

### 4.3. Statistical Analysis

Statistical analyses were done in R (version 3.0.1) [41]. Shrinkage of the tracheids in the longitudinal, tangential and radial directions of the four corewood types was examined statistically. Since the data are multivariate (three directions), we performed a two-way factorial multivariate analysis of variance (MANOVA) looking for differences between corewood types and trees. To display the differences between the centroids, we used canonical discriminant analysis (CDA) to plot the centroids in two dimensions with a minimum loss of information [19]. We also did a similar analysis to examine the shrinkage of the tracheid walls: longitudinal wall shrinkage, which is exactly the same measurement as the longitudinal shrinkage of the tracheids as a whole; transverse wall shrinkage of the radial walls, which is wall shrinkage in the tangential direction (Figure 3); and transverse wall shrinkage of the tangential walls, which is wall shrinkage in the radial direction (Figure 3). The significance of differences between corewood types in the diameters of tracheid bordered pits and their apertures and the changes in these caused by going from RH 100% to 10% were investigated using one-way analysis of variance (ANOVA). The post hoc Duncan’s multiple range test [42] was then used to determine the significance of differences between pairs of corewood types. The significance of difference between corewood types and between trees in the thicknesses of the radial and tangential walls and in the lumen diameters in the radial and tangential directions were investigated using three-way analysis of variance (ANOVA).

The multivariate approach of canonical correlation analysis [41] was performed to investigate possible relationships between shrinkage of the tracheids and the tracheid cell-wall matrix compositions of the four corewood types. Two sets of random variables were involved, set 1 represented the shrinkage variables (longitudinal, tangential and radial) and set 2 represented the chemical composition variables (lignin content and the non-cellulosic neutral monosaccharide compositions obtained by acid hydrolysis) [12,13] (Table S2). Three pairs of canonical variables were defined. The overall canonical correlation analysis provided an overview of the relationship between two groups of variables and the follow-up regression analysis tested the relative importance of variables in each set. A similar correlation analysis was also performed to investigate possible relationships between tracheid wall shrinkage (longitudinal wall shrinkage and transverse wall shrinkage of the radial and tangential walls). The longitudinal wall shrinkage is, of course, the same as the longitudinal tracheid shrinkage.

## 5. Conclusions

Our VP-SEM study of radiata pine corewood types showed that the tracheids in discs of MCW exhibited longitudinal shrinkage and swelling intermediate between that found in SCW and OW, with the dimensional changes increasing with CW severity. A statistical positive association was found between the galactosyl content of the wall-matrix and longitudinal shrinkage of CWs. Where MCWs occurs adjacent to OW or NW, the differences in longitudinal shrinkage and swelling between the two corewood types may thus result in warping similar to that reported when SCW and OW or NW are adjacent. Tangential and radial shrinkage of the tracheids also occurred on drying, with the tangential shrinkage being greater than radial shrinkage for all corewood types. However, unlike longitudinal shrinkage, tangential and radial shrinkage decreased with CW severity.

## Figures and Tables

**Table 1:** Average ^a^ shrinkage of tracheids in three directions for all four corewood types of three trees.

Tree	Wood Types	Longitudinal Shrinkage %	Tangential Shrinkage %	Radial Shrinkage %
**Tree 1 ^b^**	**OW**	0.99 ± 0.03	6.48 ± 0.08	3.21 ± 0.02
	**MCW1**	1.85 ± 0.07	5.64 ± 0.05	2.94 ± 0.05
	**MCW2**	2.71 ± 0.02	5.22 ± 0.03	2.23 ± 0.05
	**SCW**	3.82 ± 0.16	4.09 ± 0.04	2.08 ± 0.03
**Tree 2**	**OW**	0.89 ± 0.06	6.66 ± 0.07	3.09 ± 0.05
	**MCW1**	2.12 ± 0.05	5.81 ± 0.07	2.62 ± 0.04
	**MCW2**	2.97 ± 0.07	5.18 ± 0.06	2.30 ± 0.06
	**SCW**	3.89 ± 0.08	4.24 ± 0.04	2.10 ± 0.03
**Tree 3**	**OW**	0.92 ± 0.07	6.76 ± 0.06	3.22 ± 0.05
	**MCW1**	1.93 ± 0.05	5.89 ± 0.05	2.89 ± 0.02
	**MCW2**	2.78 ± 0.06	5.21 ± 0.04	2.51 ± 0.03
	**SCW**	3.72 ± 0.07	4.15 ± 0.04	2.16 ± 0.03

^a^ 18 tracheids were measured (means ± standard errors). ^b^ Both longitudinal shrinkage and swelling were determined for tracheids of Tree 1.

**Table 2:** Canonical discriminant analysis (CDA) structure coefficients for the first two canonical variates of the correlations between the shrinkage of tracheids and between the shrinkage of the tracheid walls in three directions, in the four corewood types of the three trees.

	CV1	CV2
**Tracheid shrinkage**		
^a^ Longitudinal tracheid shrinkage (%)	0.99	0.016
Tangential tracheid shrinkage (%)	−0.99	−0.148
Radial tracheid shrinkage (%)	−0.97	0.213
**Tracheid wall shrinkage**		
^a^ Longitudinal shrinkage (%) of tracheid walls	1.00	0.028
Transverse shrinkage (%) of tangential tracheid walls	−0.88	0.333
Transverse shrinkage (%) of radial tracheid walls	−0.913	−0.143

^a^ The longitudinal tracheid shrinkage (%) is the same as the longitudinal wall shrinkage (%).

**Table 3:** Correlations between the tracheid shrinkage and chemical variables and between the tracheid wall shrinkage and chemistry variables and their canonical variates.

	CV1	CV2	CV3
**Tracheid shrinkage variables**			
Longitudinal tracheid shrinkage %	−0.994	−0.091	0.068
Tangential tracheid shrinkage %	0.992	0.045	0.119
Radial tracheid shrinkage %	0.969	−0.189	−0.159
**Chemical variables**			
AcBr-soluble lignin ^a^	−0.985	−0.043	−0.134
Arabinose ^b^	0.902	0.229	−0.306
Galactose ^b^	−0.981	−0.029	0.181
Xylose ^b^	0.953	0.014	−0.191
Mannose ^b^	0.974	−0.156	−0.148
**Tracheid wall shrinkage variables**			
Longitudinal shrinkage (%) of tracheid walls	−0.992	0.046	−0.121
Transverse shrinkage (%) of tangential tracheid walls	0.952	−0.286	−0.105
Transverse shrinkage (%) of radial tracheid walls	0.997	0.059	−0.044
**Chemical variables**			
AcBr-soluble lignin ^a^	−0.981	0.172	−0.077
Arabinose ^b^	0.955	0.226	−0.133
Galactose ^b^	−0.990	−0.067	0.074
Xylose ^b^	0.974	0.021	−0.190
Mannose ^b^	0.975	−0.049	−0.103

^a^ Lignin content of the four corewood types have been determined by the acetyl bromide assay [13]. ^b^ The neutral monosaccharide compositions of the four corewood types have been determined by 2 M trifluoroacetic acid (TFA) hydrolysis [12].

**Table 4:** Diameters of the tracheid bordered pits and their apertures in the four corewood types of Tree 1 before and after drying (100% and 10% RH).

Wood Types	Diameters of Bordered Pits (μm) ^a^			Width of Single Border ^b^ (μm)	Diameters of Apertures (μm) ^a^		
	100% RH	10% RH	Dimensional Changes %		100% RH	10% RH	Dimensional Changes %
**OW**	10.87 ± 0.22	10.86 ± 0.22	−0.10 ± 0.11	2.81	5.25 ± 0.10	5.17 ± 0.10	−1.61 ± 0.15
**MCW 1**	10.89 ± 0.12	10.87 ± 0.12	−0.16 ± 0.14	2.81	5.28 ± 0.11	5.16 ± 0.11	−2.41 ± 0.13
**MCW 2**	10.17 ± 0.27	10.17 ± 0.27	0.01 ± 0.09	2.455	5.26 ± 0.11	5.11 ± 0.11	−2.85 ± 0.13
**SCW**	8.72 ± 0.20	8.71 ± 0.21	−0.10 ± 0.26	2.015	4.69 ± 0.13	4.47 ± 0.12	−4.69 ± 0.21

^a^ Diameters of 30 bordered pits (means ± standard errors). ^b^ By subtracting aperture diameter from pit diameter and dividing by two.

**Table 5:** One-way ANOVA followed by the post-hoc Duncan multiple range test set at 95% significance level of the diameters of tracheid bordered pits and their apertures in the four corewood types of Tree 1 before and after drying (100% and 10% RH).

	*p* Value of One-Way ANOVA	Post Hoc Contrasts by Duncan					
		OW-MCW1	OW-MCW2	MCW2-MCW1	SCW-OW	SCW-MCW1	SCW-MCW2
**Diameters of bordered pits**	*p* < 0.001 ***	*p* > 0.1	*p* < 0.05 *	*p* < 0.05 *	*p* < 0.001 ***	*p* < 0.001 ***	*p* < 0.001 ***
**Change of bordered pit diameters on drying**	*p* > 0.1	*p* > 0.1	*p* > 0.1	*p* > 0.1	*p* > 0.1	*p* > 0.1	*p* > 0.1
**Diameters of pit apertures**	*p* < 0.001 ***	*p* > 0.1	*p* > 0.1	*p* > 0.1	*p* < 0.001 ***	*p* < 0.001 ***	*p* < 0.001 ***
**Change of pit aperture diameters on drying**	*p* < 0.001 ***	*p* < 0.001 ***	*p* < 0.001 ***	*p* < 0.05 *	*p* < 0.001 ***	*p* < 0.001 ***	*p* < 0.001 ***

Significant levels: *** 0.001 ** 0.01 * 0.05.

**Table 6:** VP-SEM water vapour pressures used to obtain specific % relative humidities at 2 °C [39].

Steps	Water Vapour Pressure (Torr)	% Relative Humidity
1 and 19	5.3	100
2 and 18	4.7	90
3 and 17	4.2	80
4 and 16	3.7	70
5 and 15	3.2	60
6 and 14	2.6	50
7 and 13	2.1	40
8 and 12	1.6	30
9 and 11	1.1	20
10	0.5	10

N.B. Steps 1–10 are desorption steps. Steps 11–19 are adsorption steps.

## Supplementary Materials

The following are available online at http://www.mdpi.com/2223-7747/7/1/14/s1, Table S1: Dimensional changes ^a^ on drying of tracheid lumen diameter and wall thickness ^b^ of the four corewood types. Table S2: The chemical data for the four corewood types of three trees used for the multivariate approach of canonical correlation analysis. Figure S1: A diagram showing the preparation of a trimmed disc (100 µm tangential × 200 µm longitudinal × 500 µm radial) from an untrimmed (“as cut”) disc (0.5 mm diameter). Figure S2: VP-SEM micrographs showing the cut radial-longitudinal surfaces of a trimmed disc of SCW from Tree 1. (a) at 100% RH. (b) at 10% RH. The tracheid bordered pits (BP) are on the radial-longitudinal walls. The longitudinal shrinkage of the tracheid (T) (and wall) was determined by measuring the distance (d) between two reference points (edges of bordered pit apertures (PA) (yellow line) at each RH. B = border. Scale bar = 50 µm. Figure S3: Diagram showing the measurement of the longitudinal shrinkage of a tracheid. The distance (d) between the two referencing points (edges of bordered pit apertures) was measured. B = Pit border; BP = bordered pit; PA = pit aperture; T = tracheid. Figure S4: A diagram showing how bordered pit diameters (bpd) (red arrowed line) and bordered pit aperture diameters (pad) (black arrowed line) were measured before and after drying. B = border. T = torus. M = margo.
