# Supplementary material for: Dimensional Changes of Tracheids during Drying of Radiata Pine (Pinus radiata D. Don) Compression Woods: A Study Using Variable-Pressure Scanning Electron Microscopy (VP-SEM)
Source: Plants (Basel). 2018 Feb 27;7(1):14. doi: 10.3390/plants7010014 (PMC5874603; doi:10.3390/plants7010014)
Supplement: Supplementary file 1 [file plants-07-00014-s001.pdf]

Table S1. Dimensional changes[a] on drying of tracheid lumen diameter and wall thickness[b] of the four wood types

Tree	Wood types	Lumen diameter (μm)						Wall thickness (μm)					
		Radial			Tangential			Radial			Tangential		
		100 % RH	10 % RH	Shrinkage %	100 % RH	10 % RH	Shrinkage %	100 % RH	10 % RH	Shrinkage %	100 % RH	10 % RH	Shrinkage %
**Tree1**	**OW**	27.81± 0.45	27.38 ± 0.44	1.55 ± 0.07	24.24 ± 0.57	23.73 ± 0.56	2.08 ± 0.10	4.35 ± 0.21	4.14 ± 0.20	4.72 ± 0.18	4.46 ± 0.17	4.36 ± 0.17	2.14 ± 0.12
	**MCW1**	26.86 ± 0.59	26.32 ± 0.58	1.02 ± 0.03	23.99 ± 0.94	23.58 ± 0.93	1.75 ± 0.11	4.67 ± 0.19	4.51 ± 0.19	3.36 ± 0.18	4.56 ± 0.15	4.49 ± 0.15	1.76 ± 0.15
	**MCW2**	24.56 ± 0.61	24.34 ± 0.61	0.91 ± 0.06	22.10 ± 0.68	21.87 ± 0.67	1.02 ± 0.06	4.82 ± 0.12	4.69 ± 0.12	2.88 ± 0.13	5.21 ± 0.16	5.14 ± 0.16	1.27 ± 0.08
	**SCW**	23.15 ± 0.49	22.99 ± 0.49	0.71 ± 0.04	20.60 ± 0.73	20.46 ± 0.72	0.69 ± 0.03	5.40 ± 0.15	5.29 ± 0.15	2.02 ± 0.19	5.29 ± 0.22	5.24 ± 0.22	0.92 ± 0.06
**Tree2**	**OW**	27.68 ± 0.61	27.28 ± 0.60	1.43 ± 0.08	22.61 ± 0.57	22.22 ± 0.56	1.74 ± 0.10	4.36 ± 0.16	4.15 ± 0.15	4.89 ± 0.13	4.32 ± 0.11	4.23 ± 0.11	2.27 ± 0.09
	**MCW1**	26.46 ± 0.52	25.94 ± 0.51	0.98 ± 0.03	22.23 ± 0.81	21.87 ± 0.79	1.60 ± 0.07	4.34 ± 0.18	4.20 ± 0.18	3.34 ± 0.11	4.59 ± 0.12	4.50 ± 0.12	1.81 ± 0.08
	**MCW2**	24.62 ± 0.56	24.39 ± 0.56	0.94 ± 0.06	22.15 ± 0.92	21.86 ± 0.91	1.32 ± 0.08	4.59 ± 0.09	4.45 ± 0.08	2.91 ± 0.10	5.02 ± 0.17	4.95 ± 0.17	1.38 ± 0.08
	**SCW**	22.50 ± 0.57	22.35 ± 0.57	0.70 ± 0.04	20.72 ± 0.49	20.53 ± 0.48	0.89 ± 0.05	4.79 ± 0.23	4.69 ± 0.22	2.07 ± 0.15	5.64 ± 0.25	5.58 ± 0.25	1.06 ± 0.09
**Tree3**	**OW**	27.37 ± 0.58	26.96 ± 0.56	1.51 ± 0.08	23.88 ± 0.62	23.39 ± 0.62	2.05 ± 0.12	4.41 ± 0.19	4.21 ± 0.18	4.62 ± 0.18	4.09 ± 0.13	4.01 ± 0.13	2.00 ± 0.10
	**MCW1**	27.20 ± 0.60	26.65 ± 0.60	1.03 ± 0.04	26.50 ± 0.58	26.12 ± 0.56	1.42 ± 0.10	4.68 ± 0.15	4.51 ± 0.15	3.64 ± 0.19	4.77 ± 0.16	4.68 ± 0.15	1.97 ± 0.10
	**MCW2**	22.08 ± 0.86	21.88 ± 0.86	0.90 ± 0.05	23.15 ± 0.96	22.93 ± 0.96	0.94 ± 0.05	4.73 ± 0.15	4.60 ± 0.15	2.72 ± 0.12	4.93 ± 0.13	4.86 ± 0.13	1.44 ± 0.07
	**SCW**	21.85 ± 0.65	21.70 ± 0.65	0.67 ± 0.03	20.55 ± 0.77	20.37 ± 0.77	0.90 ± 0.05	5.15 ± 0.16	5.05 ± 0.16	1.90 ± 0.13	5.39 ± 0.19	5.32 ± 0.19	1.31 ± 0.07

[a] Obtained from the transverse surface.

[b] Thickness of double wall of two adjacent tracheids.

All values are means ± standard errors.

Table S2. The chemical data for the four wood types of three trees used for the multivariate approach of canonical correlation analysis.

Tree	Wood types	Lignin[a]	Arabinose[b]	Galactose[b]	Xylose[b]	Mannose[b]
Tree 1	**OW**	25.86	9.89	8.40	30.34	36.47
	**MCW1**	28.74	6.79	31.55	20.94	27.69
	**MCW2**	31.74	5.71	40.75	17.95	20.79
	**SCW**	35.14	4.89	49.72	15.31	16.11
Tree 2	**OW**	26.11	11.01	8.81	31.04	38.17
	**MCW1**	28.26	6.30	30.39	23.59	28.57
	**MCW2**	31.25	5.71	40.99	17.81	20.71
	**SCW**	35.03	4.09	50.53	14.16	18.89
Tree 3	**OW**	26.43	8.76	8.65	30.05	40.44
	**MCW1**	28.14	6.21	29.42	23.43	30.29
	**MCW2**	31.99	5.25	41.07	16.74	21.07
	**SCW**	34.46	3.47	49.93	16.64	18.36

[a] Lignin content of the four wood types were determined by the acetyl bromide assay [13].

[b] The neutral monosaccharide compositions of the four wood types were determined by 2 M trifluoroacetic acid (TFA) hydrolysis as a percentage of the total neutral monosaccharides [12]. The percentage of glucose is not given as it was not used in the statistical analysis.

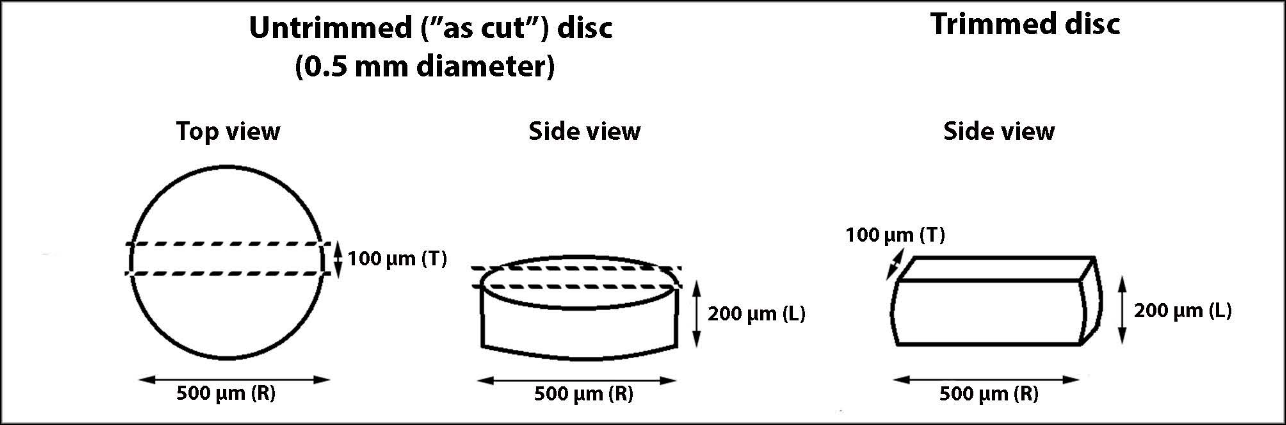
Untrimmed ("as cut") disc
(0.5 mm diameter)
Trimmed disc
Top view
Side view
Side view
100 µm (T)
200 µm (L)
100 µm (T)
200 µm (L)
500 µm (R)
500 µm (R)
500 µm (R)

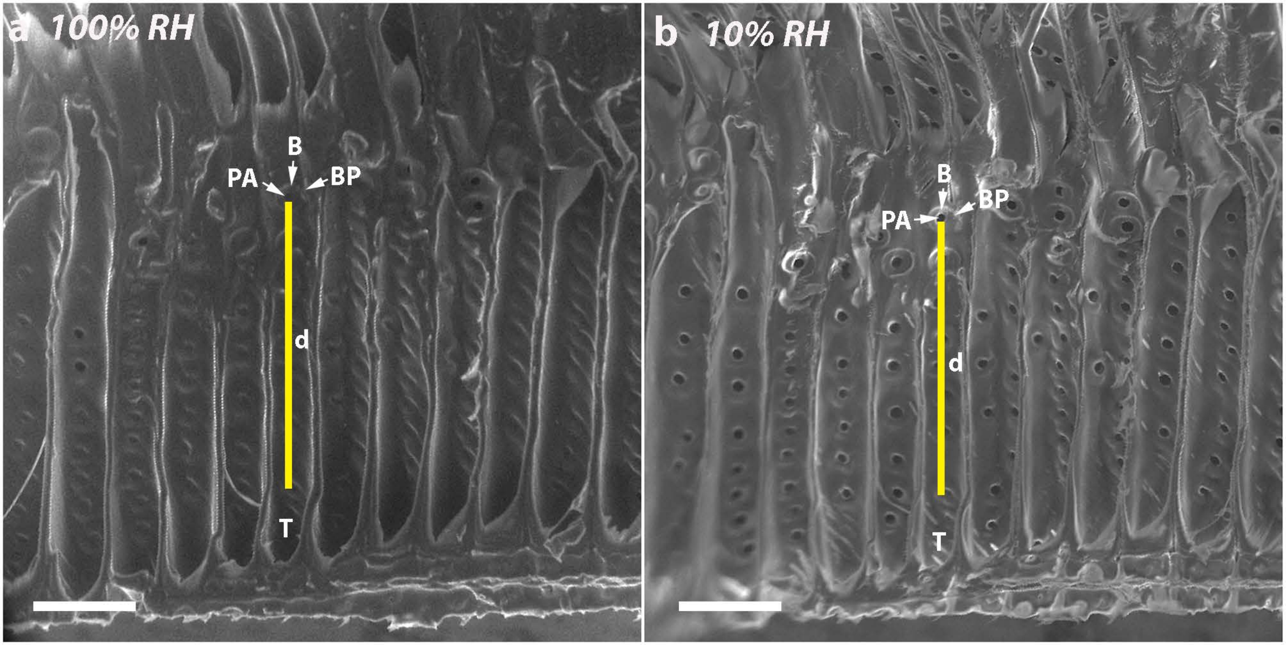

a 100% RH
B
PA
BP
d
T
b 10% RH
B
BP
PA
d
T

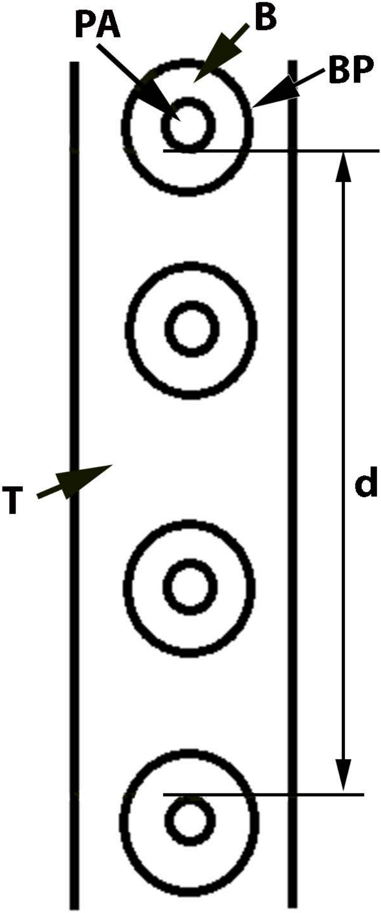
PA
B
BP
T
d

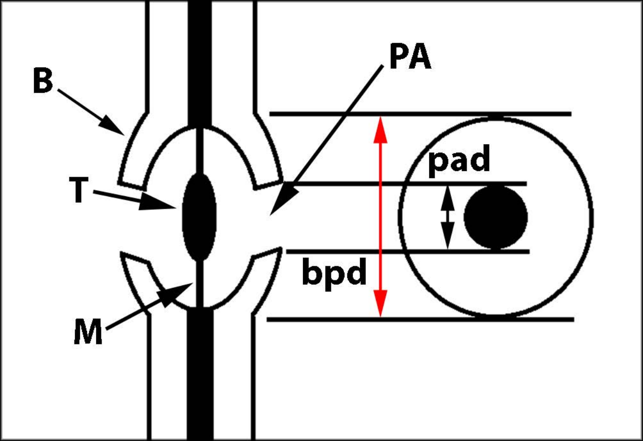
B
PA
pad
T
bpd
M
